# Simultaneous expression of epithelial and immune cell markers in circulating tumor cells identified in patients with stage 4 breast cancer

**DOI:** 10.1038/s43856-025-01024-0

**Published:** 2025-07-24

**Authors:** Nikki Higa, Audrey Limb, Valerie Hennes, Andrés Rivera, Rafael Nevarez, Anand Kolatkar, Carol K. Tweed, Adam I. Riker, Young Lee, Lorraine Tafra, Jeremy G. Perkins, Craig D. Shriver, Peter Kuhn, James Hicks

**Affiliations:** 1 Convergent Science Institute in Cancer, Michelson Center for Convergent Bioscience, University of Southern California, Los Angeles, CA 90089 USA; 2 Keck School of Medicine, University of Southern California, Los Angeles, CA 90033 USA; 3 Department of Biological Sciences, Dornsife College of Letters, Arts, and Sciences, University of Southern California, Los Angeles, CA 90089 USA; 4 Luminis Health, Anne Arundel Medical Center, DeCesaris Cancer Institute, Annapolis, MD 21401 USA; 5 Maryland Oncology Hematology, Annapolis, MD 21401 USA; 6 Precision Healthcare Specialists, Naples, FL 34102 USA; 7 Murtha Cancer Center Research Program, Department of Surgery, Uniformed Services University of the Health Sciences, Bethesda, MD 20889 USA; 8 Walter Reed National Military Medical Center, Bethesda, MD 20889 USA; 9 Institute of Urology, Catherine & Joseph Aresty Department of Urology, Keck School of Medicine, University of Southern California, Los Angeles, CA 90033 USA; 10 Norris Comprehensive Cancer Center, Keck School of Medicine, University of Southern California, Los Angeles, CA 90033 USA; 11 Department of Biomedical Engineering, Viterbi School of Engineering, University of Southern California, Los Angeles, CA 90089 USA; 12 Department of Aerospace and Mechanical Engineering, Viterbi School of Engineering, University of Southern California, Los Angeles, CA 90089 USA

**Keywords:** Tumour biomarkers, Biomarkers, Immunosurveillance

## Abstract

**Background:**

Heterogeneous circulating tumor cells (CTCs) have been implicated in the formation of new metastases. However, circulating cells expressing both tumor and immune cell proteins are often dismissed as insignificant findings in CTC studies.

**Methods:**

Two non-contemporaneous blood samples from a metastatic breast cancer patient were analyzed using an enrichment-free platform to identify canonical, epithelial-only CTCs (CD45-/cytokeratin + , epi.CTCs) and CD45 + /cytokeratin+ immune-like CTCs (im.CTCs). Single cells from both samples were subjected to copy number and protein expression profiling. A cohort of 36 metastatic breast cancer patients was then analyzed to search for additional cases with im.CTCs.

**Results:**

Here, we identified and characterized a population of CTCs exhibiting an immune-like state. In two samples from an index patient, im.CTCs outnumbered epi.CTCs, comprising >97% of the CTC population. Single-cell copy number analysis of 43 im.CTCs and 30 epi.CTCs revealed clonal alterations across both populations, confirming a shared tumor origin. Furthermore, im.CTCs contained pseudo-diploid profiles that did not reflect dilution from the addition of a normal diploid genome, indicating that they were unlikely to have originated from tumor-immune cell fusion. Protein expression analysis showed that im.CTCs express CD45 as well as other immune-related markers, such as CD3 and CD4, and the cancer stemness marker, CD44. Subsequent analysis of a metastatic breast cancer cohort identified an additional patient harboring im.CTCs with the same tumor-derived, non-fusion genome as in the index case.

**Conclusions:**

Collectively, these genomic and proteomic features distinguish im.CTCs from previously reported circulating cells may represent a novel form of tumor cell plasticity.

## Plain language summary

Tumor cells are known to take on features that allow them to survive and move to new sites. This variation can make it difficult to distinguish them from other cells in the blood. Using a platform to profile rare cells in blood samples, we identified a population of cells expressing cancer and immune cell proteins in a breast cancer patient. Genomics data confirmed that these cells originated from the tumor and that they were different from another cell type sharing a similar protein expression pattern. We analyzed additional samples and found a second patient with these immune-like tumor cells. These findings support the existence of a cancer-immune state that might play a role in helping tumor cells spread.

## Abstract (web summary)

Higa et al. analyzed circulating cells expressing cancer and immune cell markers in breast cancer patients. Based on genomic and protein expression profiling, they show that the cells were unlike previously described circulating tumor cells with immune-like phenotypes because they did not appear to arise from heterotypical cell fusion.

## Introduction

Circulating tumor cells (CTCs) are an established liquid biopsy biomarker harboring tumor-derived information that can be leveraged for minimally invasive disease profiling^1^. In epithelial cancers, CTCs are conventionally defined as nucleated cells with expression of epithelial markers (e.g., cytokeratins (CK), epithelial cell adhesion molecule (EpCAM)) and lacking expression of the pan-leukocyte marker, CD45. Beyond these conventionally defined CTCs, there is a growing appreciation for the multitude of cell types that exist in the circulation of patients with cancer. Driven by multimodal analyses involving single-cell imaging, genomic profiling, and phenotypic characterization, new subtypes of CTCs exhibiting unique cell states have been identified alongside other non-malignant, somatic cells from the tumor microenvironment. These include CTCs undergoing epithelial-to-mesenchymal transition^2–4^, CTCs with stem-cell-like features^5,6^, circulating endothelial cells^2,7^, and cancer-associated macrophage-like cells^8^.

Another emerging CTC phenotype is one marked by the combination of epithelial/cancer marker expression with at least one immune cell marker, typically CD45. The leading hypothesis for the existence of so-called “double-positive” CTCs is heterotypical cell fusion of tumor cells and macrophages, where resulting hybrid cells possess additional capabilities for enhanced survival^9,10^. Early evidence for this theory was derived from rare cases of individuals who had received bone marrow transplants prior to their cancer diagnosis and harbored donor DNA in their tumor cells^11,12^. Subsequent patient studies then identified CD45 + /epithelial+ cells with expression of macrophage markers such as CD14, CD68, and CD163^9,13,14^. Two studies also attempted to confirm the tumor lineage of CD45 + /epithelial+ cells using genomic methods but were not able to identify cancer-associated alterations in the majority of these cells^9,15^.

Aside from this hypothesis, there has been little consideration for other, non-fusion mechanisms that could lead to an immune-like phenotype in tumor cells. Some possibilities include trogocytosis, or the active transfer of cellular contents via one cell “biting” fragments off another cell^16,17^, and acquisition of extracellular vesicle (EV)-derived proteins^18,19^. Alternatively, the CD45 + /epithelial+ phenotype may reflect the increased phenotypic plasticity of tumor cells, which was recently recognized as an emerging hallmark of cancer^20^. This concept can be illustrated through the process of vasculogenic mimicry, where tumor cells with stem-like capabilities mimic the expression program and functional characteristics of endothelial cells^21^. Accordingly, it is plausible that an analogous mechanism leading to an “immune mimicry” phenotype may also exist.

In this study of a metastatic breast cancer patient, we present genomic and protein expression evidence for a distinct CTC population expressing CD45 as well as a suite of immune cell markers, including T-cell lineage markers, CD3 and CD4, and the “stem-like” and cell surface adhesion protein, CD44. These “immune-like” CTCs (im.CTCs) make up over 97% of the total CTC population in two blood draws collected before and during anticancer therapy. Copy number profiling of individual im.CTCs and classical CTCs (CD45-/CK + , epi.CTCs) revealed identical genomic alterations typical of breast cancer^22,23^. We provide further genomic evidence that these im.CTCs are unlikely to have arisen by heterotypical cell fusion by showing that they maintain the characteristics of a pseudo-diploid genome undiluted by the addition of a normal diploid genome, which would be expected in the case of fusion between a tumor and an immune cell. We then looked at baseline samples from 36 additional metastatic breast cancer patients and identified two patients with im.CTC candidates. In one of these patients, copy number analysis confirmed the presence of clonal alterations with no evidence of a fusion genome, indicating a second instance of im.CTCs. Although we do not know the mechanism through which this im.CTC population arose, the proteomic expression profile could reflect a stable state change that is capable of extended proliferation.

## Methods

### Clinical information

The index patient and subsequent patients included in the cohort analysis were enrolled as part of the BloodPAC-007 study^24^. This study enrolled breast cancer patients aged 18 or older with metastatic disease who were starting a new line of therapy at the Walter Reed National Military Medical Center Murtha Cancer Center (WRNMMC MCC) or Anne Arundel Medical Center (AAMC; Supplementary Table 1). Approval for this study was granted by the Institutional Review Board (or Ethics Committee) of the WRNMMC (WRNMMC-2018-0130), AAMC (AAMC-1109045), and University of Southern California (UP-17-00882). Written informed consent was obtained from all study participants.

### Blood processing

Peripheral blood was collected in 10 mL Cell-free DNA blood collection tubes (Streck) and shipped to USC for liquid biopsy analysis. Sample processing was carried out as previously described^25^. Briefly, blood was centrifuged to separate the plasma and cellular fraction, then subjected to ammonium chloride erythrocyte lysis to obtain nucleated cells. Nucleated cells were plated onto custom glass microscopy slides (Marienfeld) at a density of approximately 3 million cells per slide. Slides were then cryobanked at −80 °C until further use.

### Immunofluorescence staining

For the index patient, two slides from each blood draw were stained with immunofluorescent antibodies for CTC detection based on the previously reported High-Definition Single-Cell Assay (HDSCA) workflow^25,26^. One set of slides (one from each study visit) was stained with a primary antibody mixture of an anti-human CK 1,4,5,6,8,10,13,18,19 mouse IgG1/IgG2a monoclonal antibody cocktail (Sigma; Cat# C2562; Clones: C-11, PCK-26, CY-90, KS-1A3, M20, A53-B/A2; working concentration: 210 μg/mL), an anti-human CK 19 mouse IgG1 monoclonal antibody (Dako; Cat# GA61561–2; Clone: RCK108; working concentration: 0.2 μg/mL), an anti-human CD45:Alexa Fluor 647 mouse IgG2a monoclonal antibody (AbD Serotec; Cat# MCA87A647; Clone: F10–89–4; working concentration: 1.6 μg/mL), and an anti-human CD41 rabbit IgG polyclonal antibody (Invitrogen; Cat# PA522307; working concentration: 2.5 μg/mL) followed by a secondary antibody mixture of an anti-mouse IgG1:Alexa Fluor 555 goat IgG polyclonal antibody (Invitrogen; Cat# A21127; working concentration: 4 μg/mL), anti-rabbit IgG:Alexa Fluor 488 goat IgG polyclonal antibody (Abcam; Cat# ab150077; working concentration: 4 μg/mL), and 40,6-diamidino-2-phenylindole (DAPI) for nuclear DNA (Thermo Fisher Scientific; Cat# D1306; Dilution: 1:50,000). This set was subsequently used for the copy number profiling experiments. A second set of slides (one from each study visit) was also stained as described above, minus the CD41 primary and Alexa 488 secondary antibodies. This second set was used for the targeted proteomics experiments.

### Immunofluorescence image analysis

After staining, slides were mounted and scanned as previously described^27^. Automated fluorescence scanning microscopy was used at 100x magnification to produce 2304 images per slide. A published analysis pipeline using the EBImage v4.12.2 package^28^ in R was applied to segment images, extract cell features, and identify rare cell candidates^27^. A mean CK signal greater than 6 standard deviations above the slide-wide mean was set as the threshold to classify cells as CK + . To differentiate between CD45 + /CK+ and CD45-/CK+ cells, a logistic regression classifier was developed using a subset of 320 human-annotated cell images and mean CD45 intensity as input. The model was trained on two-thirds of this dataset with 5-fold cross validation repeated 5 times using the caret v6.6-92 package^29^ in R. The resulting model correctly classified all cells in the test set and was subsequently applied to determine CD45 positivity in the remaining 5092 CK+ cells. Once cells were classified as CD45 + /CK+ or CD45-/CK + , candidates for downstream copy number or proteomic analysis were re-imaged using a fluorescence microscope at 400x magnification. The ImageJ software^30^ was used to apply linear brightness/contrast adjustments to the entire frame and generate multi-color composites for publication.

### Copy number alteration analysis

Single cells were isolated by micromanipulation and subjected to an established workflow for whole-genome copy number profiling^26,31^. Cells were lysed, and whole-genome amplification and library preparation were performed using the WGA4 Genomeplex Single-Cell Whole-Genome Amplification Kit (Sigma-Aldrich; Cat#: WGA4) and NEBNext Ultra DNA Library Prep Kit (New England Biolabs; Cat#: E7370), respectively. Libraries were sequenced on an Illumina HiSeq 4000 instrument by Fulgent Genomics (Temple City, CA) to generate 150-base-pair paired-end reads. Sequencing reads were mapped to the hg19 human reference genome using BWA-MEM v0.7.17^32^ then duplicate and low-quality reads were removed. Read counts were determined for ~5000 variable-length bins and normalized for GC-content. GC-corrected read counts were then normalized as ratios to the genome-wide mean, and profiles were segmented using circular binary segmentation^33^.

### Imaging mass cytometry

One slide from each study visit was subjected to targeted proteomic analysis by imaging mass cytometry (IMC), as previously described^34^. Slides were stained with a cocktail of metal-conjugated antibodies as well as a DNA intercalator (Supplementary Table 2). Antibodies that were not pre-labeled with a metal tag were custom-conjugated in the lab using the Maxpar antibody labeling kit (Standard BioTools). Regions of interest (ROIs) measuring 400 × 400 μm were ablated, and isotope signals were detected and quantified using a CyTOF Helios instrument (Standard BioTools). Ion count data were used to construct images at 1 μm^2^ resolution for each metal. Segmentation was carried out with a pipeline developed by the Bodenmiller Lab^35^.

After segmentation, im.CTCs and epi.CTCs were identified by matching their locations from the immunofluorescence images. The remaining masks were filtered for areas between 40-200 pixels and DNA1 ion count greater than or equal to 4 to remove poorly segmented and/or non-nucleated objects. Signal-to-noise profiles were also assessed for each marker by comparing the distributions of background ion counts and cellular ion counts. This resulted in 22 markers plus the DNA intercalator for analysis. Manual gating was performed on ion count data using the leukocyte lineage markers CD45, CD3, CD14, CD20, CD56, and CD68, plus CK8/18 as a negative exclusion marker (Supplementary Fig. 1a). Backgating was performed to refine these populations, after which CD4 and CD8 gating was performed on CD3+ cells. Manually gated populations were also compared to clusters identified using the Phenograph algorithm^36^ v0.99.1, implemented in R (Supplementary Fig. 1b). Phenograph clustering was performed on ion count data normalized to the 99^th^ percentile with default parameters. Ion counts were arcsinh transformed with a cofactor of 1 for visualization.

### Cohort analysis

For the cohort analysis, the first study drew from 36 additional metastatic breast cancer patients in the BloodPAC-007 study was analyzed. From the previous study, two slides from each draw had been stained with an updated version of the HDSCA assay, which includes CD31 (BioRad; Cat# MCA178A647; Clone: WM59; working concentration: 2.5 μg/mL) in the same fluorescence channel as CD45^27^. Immunofluorescence images were processed through the same pipeline described above to identify CK+ cells that were also positive in the CD45/CD31 channel. For samples with CK + , CD45/CD31+ cells, an additional slide was then stained with the assay used for the index patient to identify CK + /CD45+ cells.

### Data visualization and statistical analysis

Data visualization and statistical testing were performed in R v4.1.2. Two-sided Student’s t-test was used to compare *PTPRC* copy number ratios between im.CTCs and epi.CTCs. Plots were created with the ggplot2 v3.3.6^37^, ComplexHeatmap v2.10.0^38,39^, and umap v0.2.10.0 packages.

### Reporting summary

Further information on research design is available in the Nature Portfolio Reporting Summary linked to this article.

## Results

### Clinical information and analysis workflow

**Fig. 1:**
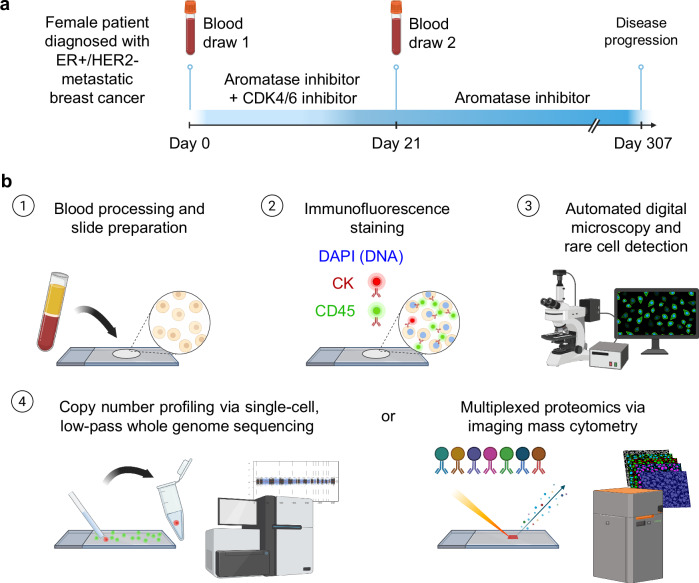
Case study overview. **a** Patient’s clinical and sample collection timeline. **b** Overview of the HDSCA workflow. Following blood processing and cell plating, slides are stained with immunofluorescent antibodies and imaged to identify candidate cells for downstream analysis. Slides can either be used for single-cell copy number analysis or multiplexed proteomics profiling.

The female patient was diagnosed with estrogen receptor (ER) positive, HER2 negative, lobular breast cancer that had metastasized to the skin and bone. The patient was started on a combination of letrozole and palbociclib but experienced disease progression after approximately 10 months. Peripheral blood samples were collected for liquid biopsy analysis prior to and after three weeks of first-line therapy (Fig. 1a). Slides prepared from each blood draw were stained with immunofluorescence assays to detect CTCs. Cells were then subjected to either whole-genome copy number analysis or targeted proteomic profiling (Fig. 1b).

### CD45 + CTCs greatly outnumber CD45- CTCs

**Fig. 2:**
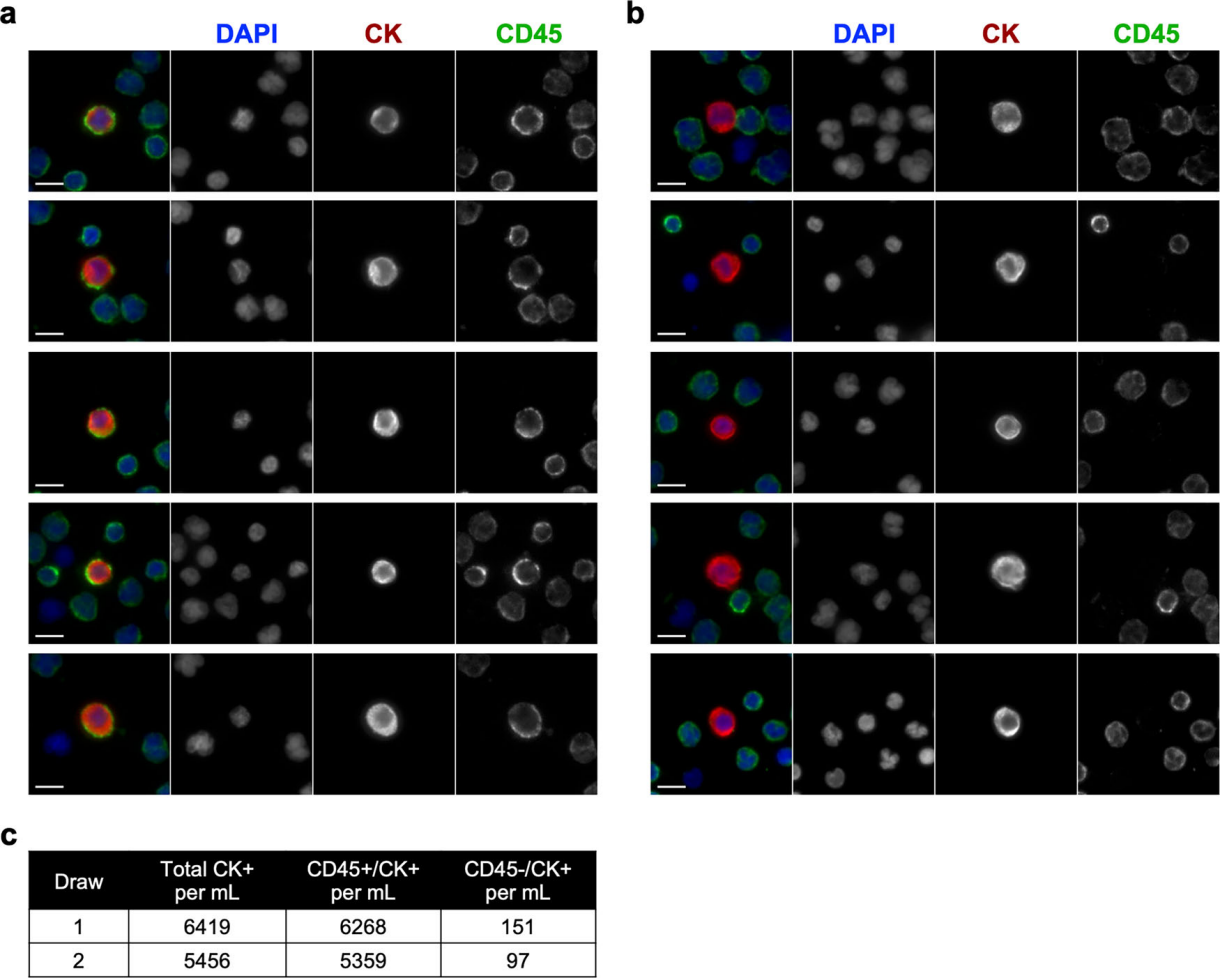
CD45+ and CD45- CTCs were detected in the index patient. **a** CD45+ CTCs and **b** classical CD45- CTCs. Composite images are displayed to the left of the individual channel images (color code: blue = DAPI, red = CK, green = CD45; scale bar = 10 μm).** c** Enumeration of CD45+ and CD45- CTCs from two serial blood draws.

In samples from the two timepoints, a large population of cells (>5000 cells per mL of blood) with CK expression characteristic of a CTC was detected. Within this population of CTC candidates, we observed cells that were also positive for the pan-leukocyte marker, CD45 (Fig. 2a, b). After classifying the CK+ cells as CD45+ or CD45-, we found that 97% were CD45+ and that this was consistent between the two samples (6268/6419 and 5359/5456 CK+ cells per mL for the first and second draw, respectively; Fig. 2c).

### im.CTCs and epi.CTCs contain identical tumor genomes

**Fig. 3:**
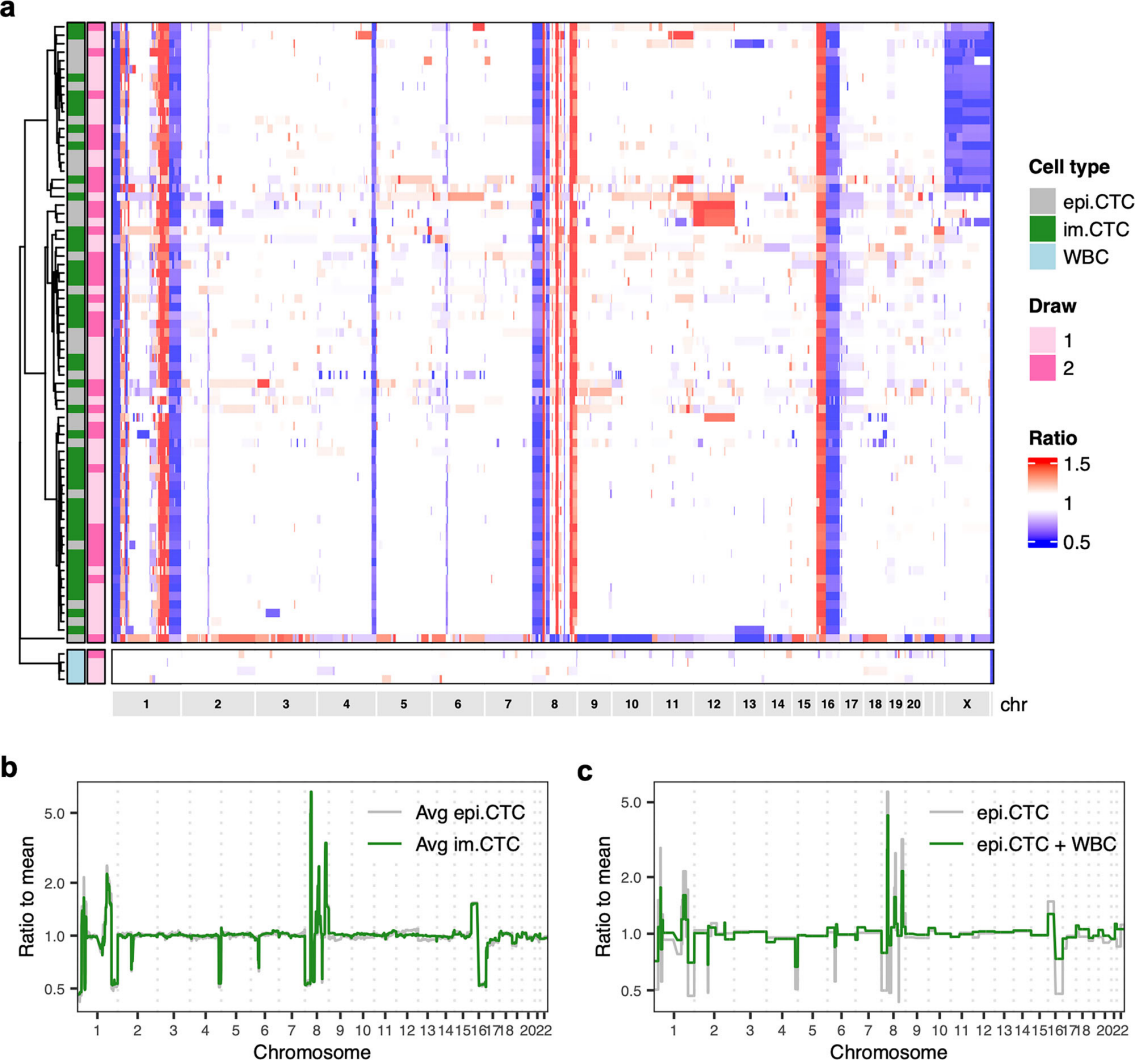
Copy number analysis of im.CTCs and epi.CTCs. **a** Heatmap of copy number profiles for 77 single cells (43 im.CTCs, 30 epi.CTCs, 4 WBCs). Copy number ratios for 5000 genomic bins spanning chromosome 1 to chromosome Y are represented by the color scale at the right, with gains in red and losses in blue. Cell type and draw number are annotated on the left side of the heatmap. **b** Overlay of averaged copy number profiles across all epi.CTCs (gray) and im.CTCs (green) sequenced. **c** Comparison of copy number profiles for an epi.CTC (gray) versus a synthetic tumor-WBC hybrid (green) formed by cell fusion. Fusion cell profile was synthetically generated by combining profiles of a single epi.CTC and a single WBC from the index patient. Bin read counts for each individual cell were summed, corrected for GC-content, and then used to determine new bin ratios for the combined profile.

To further probe the tumor lineage of CTC candidates, we performed single-cell whole-genome copy number profiling. A total of 77 cells were sequenced, including 43 CD45 + CTC candidates, 30 CD45- CTC candidates, and 4 white blood cell (WBC) controls. This approach provided important advantages for characterizing CD45 + CTC candidates. First, copy number alterations (CNAs) are a characteristic mutation of breast cancer and can be used to infer clonal structure amongst tumor cell populations^22,23,40^. In contrast to the WBC controls, nearly all CNAs were shared between CD45+ and CD45- CTC candidates including gains and losses on chromosome 1, loss on 4q, a “firestorm”-like signature^41^ on chromosome 8, 16p gain, and 16q loss (Fig. 3a). From this, we concluded that both populations of CTC candidates were indeed tumor-derived and shared a common lineage. Hereon, we refer to the CD45+ population as im.CTCs to distinguish them from the canonical CD45-epi.CTCs. Second, and most important for the argument that these profiles are not the result of fusion with a WBC, the ratios of the gains and losses in the im.CTCs were nearly identical to those in the epi.CTCs (Fig. 3b). It is well established that the copy number profiling method used here accurately reflects the integer copy number states of chromosome segments through the ratios derived from the bin read counts^31,40,42^. For example, in an initially diploid cell, a gain of one copy would result in a 3:2 ratio (+1.5) and a loss would result in a 2:1 ratio (0.5), as observed on chromosome 16 for all of the CTCs profiled (Fig. 3b). In the case of fusion with a WBC, addition of a full diploid genome would change those ratios to 5:4 ( + 1.25) and 4:3 (0.75), resulting in a “compressed” profile. To illustrate this, we combined bin counts from a single WBC and an epi.CTC to generate a synthetic fusion cell copy number profile showing marked dampening of alteration amplitudes throughout the genome (Fig. 3c). Along these lines, we find that compared to the epi.CTC profiles, im.CTC profiles did not exhibit the level of compression that would be expected for a tumor-WBC fusion genome. We did not observe changes in CNAs and alteration amplitudes between CTCs from the first and second blood draw (Supplementary Fig 2).

Other notable features of the copy number profiles included partial or complete loss of chromosome X in a fraction of both the im.CTCs and epi.CTCs (Supplementary Fig. 3a). Loss on 2q and gain of chromosome 12 were also shared by a group of three epi.CTCs, while a small number of additional gains and losses were observed in individual cells (e.g. gains on chromosomes 4, 6, and 11, and losses on chromosomes 1, 3, and 13; Fig. 3a). We note that *PTPRC*, the gene encoding for CD45, is located on chromosome 1q and that the region encompassing this gene exhibited a single copy gain in the majority of sequenced cells, however, there was no significant difference in the copy number ratio observed for im.CTCs versus epi.CTCs (Supplementary Fig. 3b, c).

### im.CTCs express additional immune-related markers

**Fig. 4:**
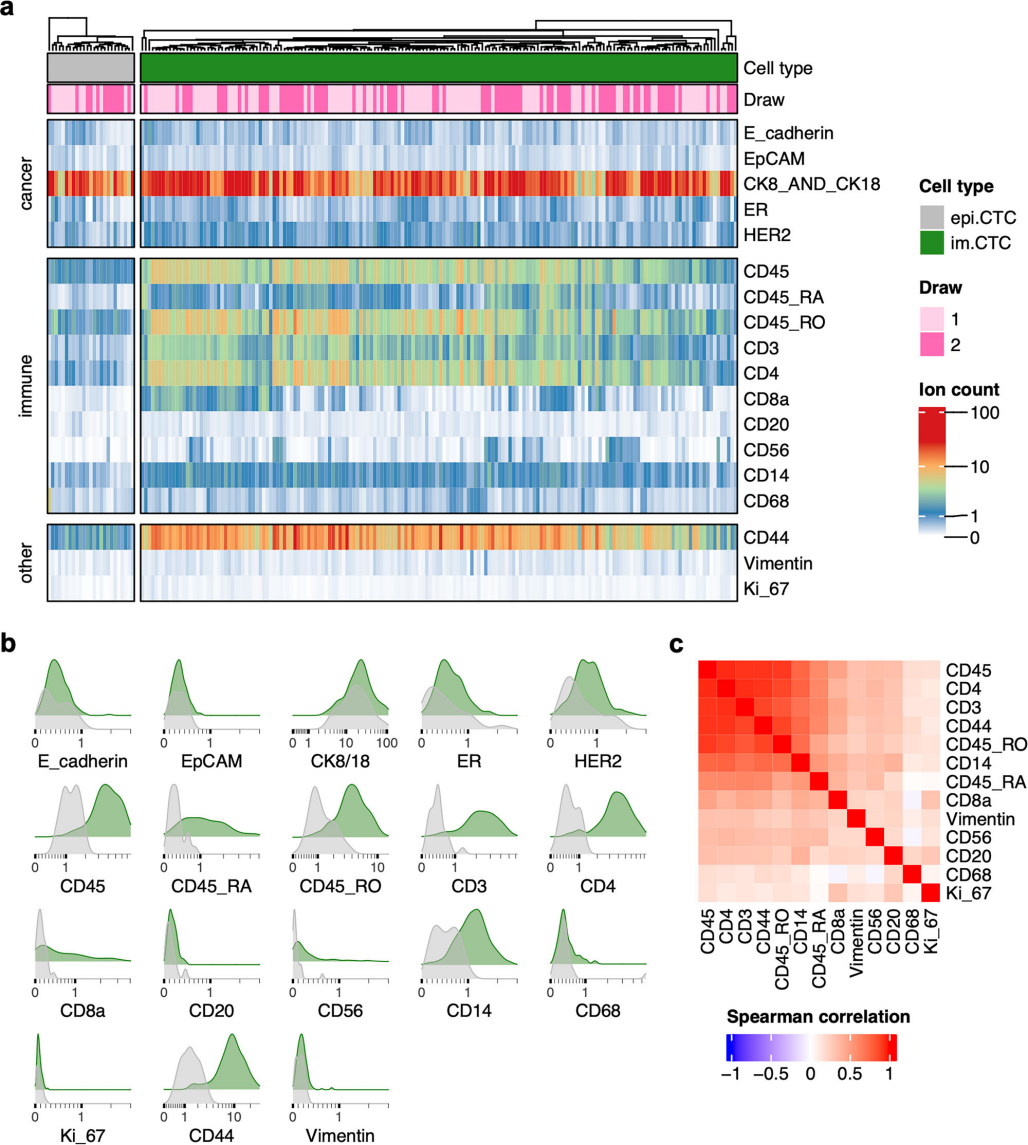
Comparison of CTC proteomic profiles. **a** Heatmap of proteomic expression profiles for epi.CTCs and im.CTCs. Cell type and draw number are annotated at the top. Arcsinh-transformed color scale was used to display ion counts. **b** Distributions of individual marker expression in epi.CTCs (gray) compared to im.CTCs (green). Ion counts are displayed with arcsinh-transformed *x*-axes. **c** Spearman correlation matrix displaying pairwise relationships between immune-related markers, CD44, vimentin, and Ki67.

Proteomic analysis using IMC was performed to explore immune-related marker expression. Across 197 CK+ cells (172 im.CTCs, 25 epi.CTCs) from both draws, im.CTCs and epi.CTCs exhibited distinct expression profiles, with multiple immune markers more highly expressed in im.CTCs (Fig. 4a, b). These included CD45, CD3, CD4, CD44, CD14, CD45RO, and to some extent, CD45RA, many of which were also strongly correlated with CD45 expression (Fig. 4c). Epithelial and breast cancer-associated markers CK8 and CK18 were highly expressed in im.CTCs and epi.CTCs, while expression of E-cadherin, EpCAM, and HER2 was relatively low (Fig. 4a, b, Supplementary Fig. 4). Given that this is a lobular breast cancer case, low expression of E-cadherin and EpCAM is not unusual^43,44^. The B-cell marker, CD20, the macrophage marker, CD68, and the epithelial-to-mesenchymal transition marker, vimentin, were lowly expressed in both cell types (Fig. 4a, b). As with the copy number profiles, we did not observe marked changes in the protein expression profiles between im.CTCs from the first and second blood draws (Supplementary Fig. 5).

**Fig. 5:**
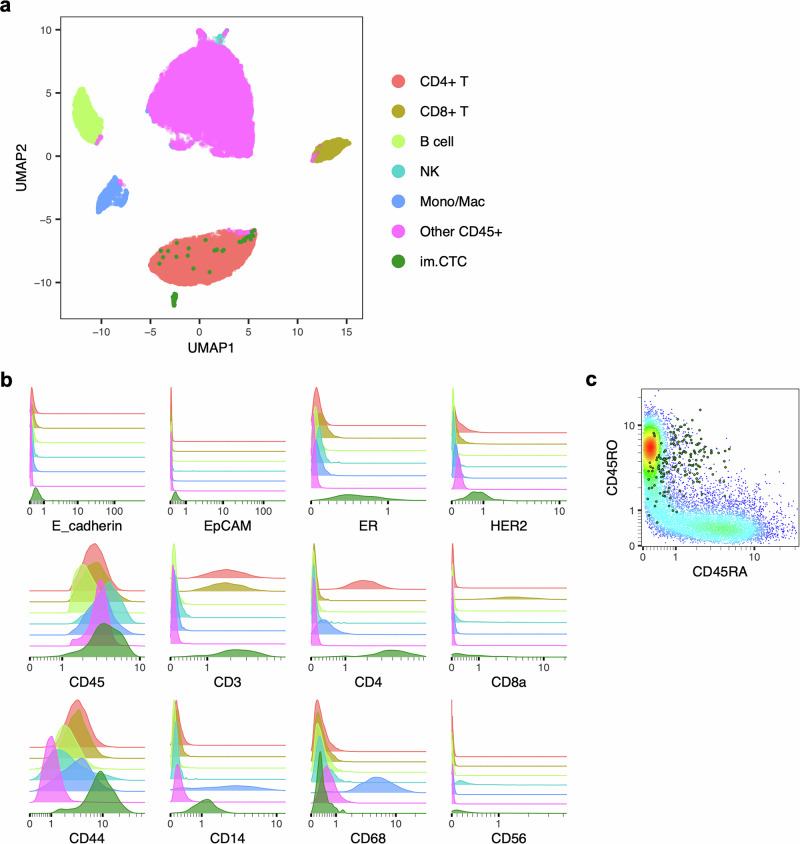
Comparison of im.CTC and WBC proteomic profiles. **a** UMAP projection constructed from immune marker expression profiles of gated WBC populations and im.CTCs. **b** Distributions of cancer and immune marker expression across WBCs compared to im.CTCs. Ion counts are displayed with arcsinh-transformed *x*-axes. Colors correspond to cell types from the legend in (**a**). **c** Scatter plot of CD45RO versus CD45RA expression in im.CTCs, indicated as green dots. The density plot in the background was generated from the CD3 + T-cell population for comparison. Ion counts are displayed with arcsinh-transformed axes.

In terms of the immune cell population (*n* = 42,005 cells), im.CTCs most closely resembled the expression profile of CD4 + T cells (Fig. 5a) and showed comparable levels of CD45 expression relative to different immune cell subsets (Fig. 5b). With respect to the monocyte/macrophage subset, im.CTCs showed moderate CD14 expression but no CD68 expression (Fig. 5b). Following up on the high T-cell marker expression seen in im.CTCs, we also found that levels of CD3 were comparable between im.CTCs and the CD3 + T-cell population, levels of CD4 in im.CTCs were slightly higher than those seen in the CD3 + /CD4 + T-cell subset, and a small proportion of im.CTCs also displayed elevated CD8 expression (Fig. 5b). CD44 was more highly expressed in im.CTCs compared to all other immune cell populations (Fig. 5b). When looking at markers for naïve versus memory T cells, im.CTCs showed higher expression of CD45RO than CD45RA but generally expressed intermediate levels of both isoforms compared to the general T-cell population (Fig. 5c).

### im.CTC candidates are identified in additional metastatic breast cancer patients

After characterizing im.CTCs from the index patient, we expanded the search to see if these cells could be found in other patients. We reanalyzed 36 samples from 36 additional metastatic breast cancer patients that were enrolled in the same original study as the index case and identified im.CTC candidates (CD45 + /CK+ cells) in two patients (Supplementary Fig. 6). Patient #2 had an im.CTC count of 35.1 cells/mL, which was equivalent to 24.6% of CTC candidates. Patient #3 had 35.9 im.CTCs/mL, comprising 27.1% of their total CTC candidates.

**Fig. 6:**
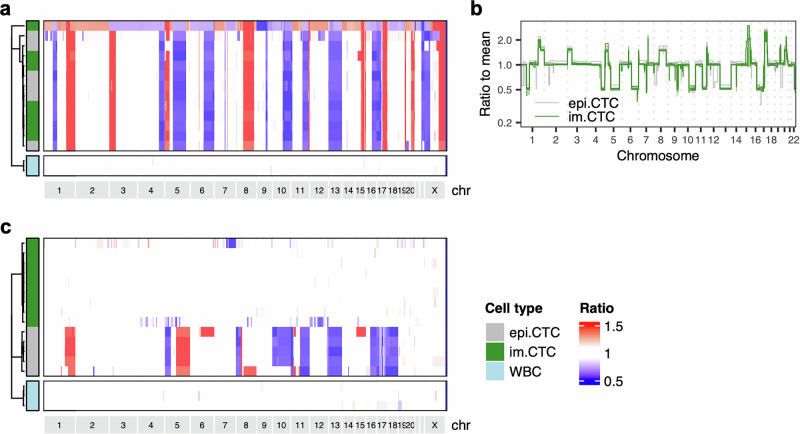
Copy number analysis of CTC candidates from two additional metastatic breast cancer patients. **a** Heatmaps of copy number profiles for 15 single cells (7 im.CTCs, 6 epi.CTCs, 2 WBCs) from patient #2. **b** Overlay of individual profiles for epi.CTC and im.CTC candidates from patient #2. One im.CTC with multiple regions of copy number loss is not shown. **c** Heatmaps of copy number profiles for 17 single cells (9 im.CTCs, 5 epi.CTCs, 3 WBCs) from patient #3.

As with the index case, im.CTC candidates from each patient were subjected to copy number profiling to evaluate clonality and evidence of cell fusion. All 7 im.CTCs sequenced from patient #2 displayed CNAs that were clonal with the epi.CTC population (*n* = 5) (Fig. 6a). Comparison of the copy number profiles also showed no compression of CNA amplitudes to indicate a fusion genome in the im.CTCs (Fig. 6b). Taken together, we reasoned that the im.CTCs from this patient represented a second instance of the CD45 + , tumor-derived, non-fusion im.CTCs observed in the index patient. In contrast, none of the im.CTC candidates (*n* = 9) from patient #3 contained clonal alterations (Fig. 6c). Non-clonal copy number losses were found in two im.CTCs, however, were detected in the absence of other CNAs that would signify a tumor lineage. Accordingly, the im.CTCs candidates from patient #3 do not appear to be cancer cells, despite their CD45 + /CK+ phenotype.

## Discussion

To our knowledge, this is the first study to report genomic evidence of tumor origin and broad immune phenotyping in im.CTCs with matched epi.CTCs from patient samples. The combination of non-fusion cancer genomes and immune-like expression profiles in these cells distinguishes them as a unique population in the milieu of circulating cells studied in the context of cancer.

Prior reports of CD45 + /CK+ cells have typically attributed them to artifacts or, more recently, as products of tumor-macrophage fusion^15,45,46^. Here, we provide genomic evidence supporting a non-fusion, tumor origin that was consistent across all im.CTCs profiled. In the early stages of cell fusion, the resulting cell retains both parent cell nuclei, as evinced by the additive ploidy values observed in hybrids formed in vitro^47,48^. We demonstrate that this addition of a non-altered, diploid WBC genome with an aneuploid tumor cell genome would result in attenuated CNA amplitudes and find that this was not the case for the im.CTCs in this study. Additionally, fusion of the parent nuclei and subsequent mitotic events occurring later in the fusion process often result in novel chromosomal aberrations not seen in the parent cells^48,49^. Meanwhile, unique CNAs were a rare occurrence across the cells we profiled. Overall, the high degree of similarity between im.CTCs and epi.CTC copy number profiles clearly indicate that these im.CTCs are bona fide tumor cells and differentiate them from hybrid cells stemming from tumor-macrophage fusion.

It is worth noting that im.CTCs from the index case exhibited moderate CD14 expression despite their non-fusion origin. By relying on a limited number of proteomic markers, studies using CD14 or even CD45 alone to identify tumor-macrophage hybrids may erroneously include non-fusion cells in their analysis. The non-cancerous im.CTC candidate cells from patient #3 further underscore the difficulty of determining the identity of CD45 + /CK+ cells based on immunofluorescence images.

Beyond CD45, im.CTC proteomic profiles from the index case revealed strong expression of the T-cell markers CD3 and CD4, as well as CD44. The co-occurrence of CD3 and CD4 expression was unexpected as neither has notable functions outside of their roles in T-cell activation. One possibility is that these T-cell surface proteins may have been acquired from T-cell-derived EVs. A limited number of studies have shown that tumor cell uptake of leukocyte surface proteins can occur through internalization of leukocyte-derived EVs^18,19,50^. One study also found that CD45 on the surface of cancer cells could dimerize with CD45 molecules on T cells and hinder TCR signaling required for T-cell-mediated killing^19^. These mechanisms have yet to be fully understood, so it is unclear if EV-mediated protein transfer can produce the high number of im.CTCs seen in these patients, and whether EV-derived CD45 can be maintained in CTCs at the levels we observed.

On the other hand, CD44 is commonly regarded as a marker of cancer stem cells (CSCs), which possess increased plasticity and self-renewal capabilities that are believed to drive disease relapse and therapy resistance^51^. Previously, a study from Park et al. identified a potential link between CD45 and the CSC state through its role in promoting β-catenin accumulation, leading to increased Wnt transcriptional activity and upregulation of stemness genes^52^. In a separate study, CD44 expression was also detected in CD45+ cells co-expressing E-cadherin and/or CK in both tissue and blood specimens from a patient with metastatic breast cancer^15^. Though it is not immediately clear why upregulation of CD3 and CD4 would be advantageous for tumor cells, these may reflect an acquired phenotype afforded by the plasticity of a CSC state. It is also important to acknowledge that while CD44 is often associated with CSCs, it is widely expressed across tissues^53^ and is known to be upregulated following T-cell activation^54,55^. Whether the CD44 expression in im.CTCs reflect a CSC-like state, or whether it is one of immune cell proteins gained through the acquisition of a T-cell-like phenotype, remains to be determined.

The notion that cancer cells may adopt features of a non-malignant, immune cell type is reminiscent of a phenomenon known as “vasculogenic mimicry”, defined as the de novo formation of vascular networks by tumor cells with upregulated endothelial-related pathways^21^. Vasculogenic mimicry was initially discovered in melanoma and has since been reported across several types of carcinomas, sarcomas, and tumors of the central nervous system^56–58^. Unsurprisingly, CSC-like features are often associated with promoting vasculogenic capabilities^59^, and it is possible that stemness may similarly enable a form of “immune mimicry” as seen in the im.CTCs.

The presence of im.CTCs in two out of the 37 (5.4%) metastatic breast cancer patients analyzed, both of which had relatively high CTC counts, suggest that the biology leading to im.CTCs occur in a relatively small fraction of patients. Due to the small sample size, we were unable to assess correlations between im.CTCs and clinical endpoints. Future studies are needed to assess the generalizability of our findings across additional cancer types and stages. Another limitation of this study was the lack of functional studies to understand the role of immune-like expression by tumor cells in the metastatic process. The sample preparation protocols used in this study required cell fixation, so viable cells were not available. Lastly, due to the large number of im.CTC candidates in the index case, we were not able to perform copy number profiling on every candidate to confirm their tumor origin. However, all 43 of the im.CTC candidates sequenced were clonally altered with no evidence of a fusion genome.

Altogether, this study provides single-cell genomic and proteomic evidence to comprehensively depict the unique cancer-immune state exhibited by im.CTCs detected from clinical samples. Unlike circulating hybrid cells described previously, the im.CTCs in this study were not consistent with a tumor-immune cell fusion origin and contained bona fide cancer genomes while displaying strong expression of multiple immune-associated markers. The non-canonical, immune-like phenotype identified in im.CTCs may mark an underappreciated mechanism supporting enhanced metastatic capabilities and warrant further investigation as a novel therapeutic and biomarker opportunity.

## Supplementary information

SUPPLEMENTAL MATERIAL

Reporting Summary

## Data Availability

The data presented in this study are openly available in the BloodPAC Commons at https://data.bloodpac.org/discovery/BPDC000144, reference number BPDC000144. Data are also available from the corresponding author on reasonable request.

## Code availability

The code used in this study utilizes standard third-party open-source libraries packaged in R and Python. The image analysis code used to identify rare cell candidates in the HDSCA workflow uses custom code and is proprietary and licensed to Epic Sciences for commercial use.
