# Supplementary material for: Simultaneous expression of epithelial and immune cell markers in circulating tumor cells identified in patients with stage 4 breast cancer
Source: Commun Med (Lond). 2025 Jul 24;5:309. doi: 10.1038/s43856-025-01024-0 (PMC12290075; doi:10.1038/s43856-025-01024-0)
Supplement: Supplementary file 1 — SUPPLEMENTAL MATERIAL [file 43856_2025_1024_MOESM1_ESM.pdf]

Supplementary Figures

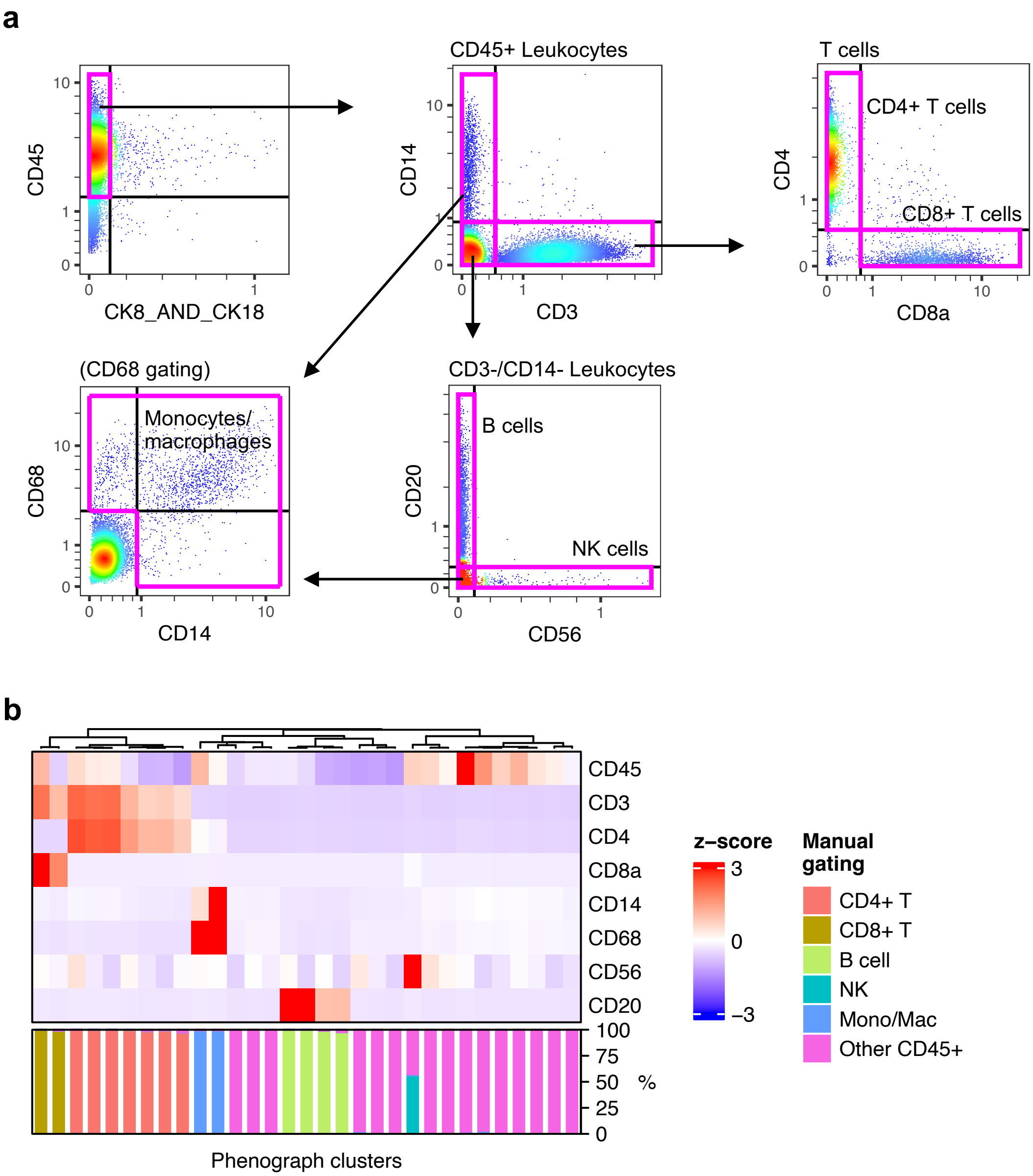

Supplementary Figure 1. **Gating strategy for IMC data.** (**a**) Overview of manual gating process. First, CD45 and CK were used to identify leukocytes (CD45+/CK-). Next, CD3 and CD14 were used to identify the T-cell (CD3+/CD14-) and monocyte/macrophage (CD3-/CD14+) populations. Cells negative for both markers were then gated on CD20 and CD56 to identify the B-cell (CD20+/CD56-) and NK cell (CD20-/CD56+) populations. Cells negative for these markers were further gated for CD68 to identify CD68+ monocytes/macrophages. CD14+ cells are included in the same plot to display the collective monocyte/macrophage population. Cells that were negative for CD68 were labeled as “Other CD45+” cells. The T-cell population was also gated on CD4 and CD8a to identify the CD4+ and CD8+ subsets. Ion counts are displayed with arcsinh-transformed axes. (**b**) Comparison of phenograph clustering with manual gating. Heatmap represents z-scored ion counts of cluster averages for markers used in immune cell type classification. Bar plot displays the percentage of manually gated populations within each phenograph-determined cluster.

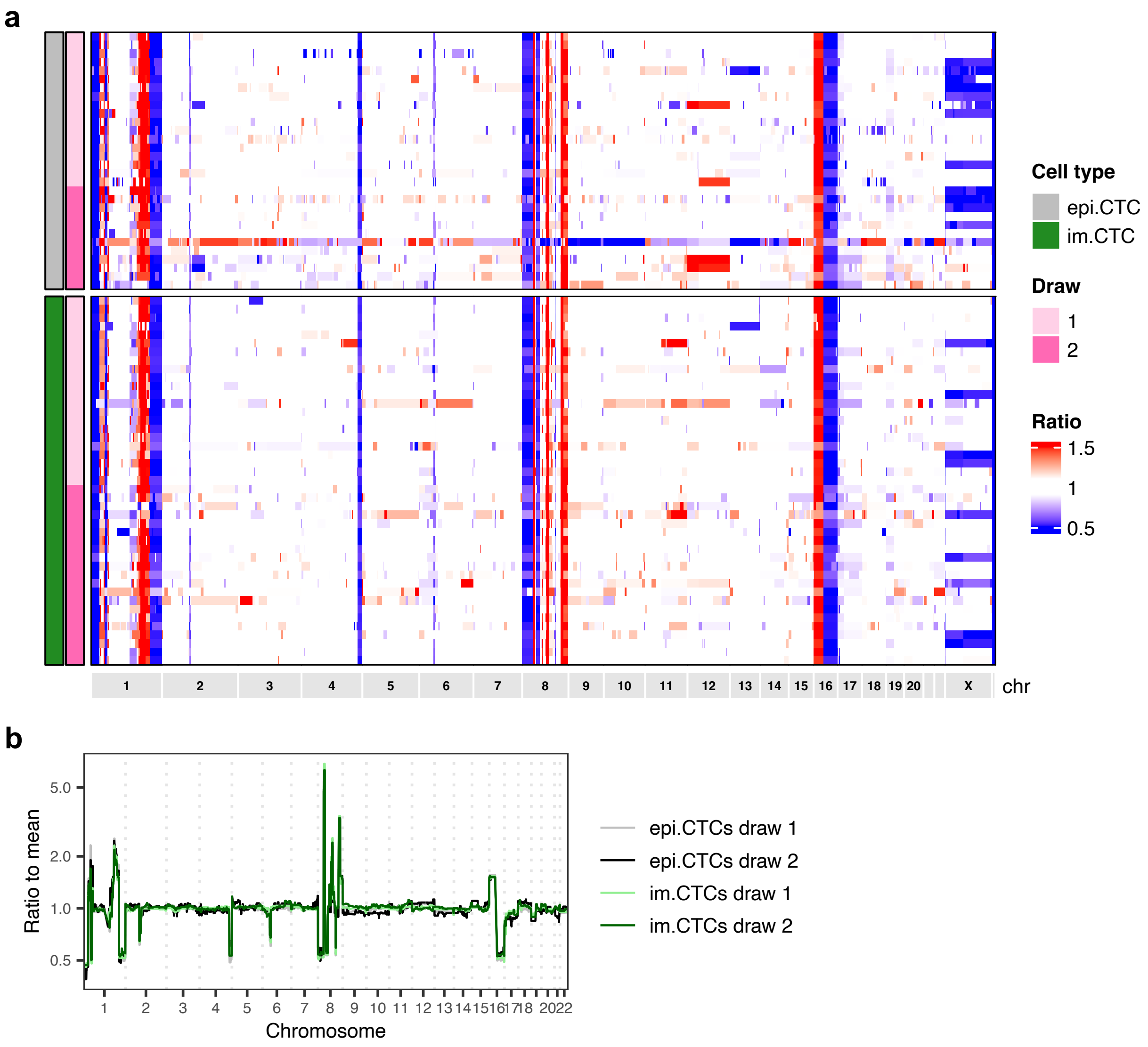

Supplementary Figure 2. **Copy number profiles for CTCs from pre- and post-treatment draws.** (**a**) Heatmap of copy number profiles for 43 im.CTCs (draw 1: n=22; draw 2: n=21) and 30 epi.CTCs (draw 1: n=18; draw 2: n=12) from the first and second blood draws. (**b**) Overlay of averaged copy number profiles across epi.CTCs and im.CTCs within each blood draw.

a

ChrX loss (N=21)	# / total (%)
epi.CTCs	12/30 (40.0)
im.CTCs	9/43 (20.9)

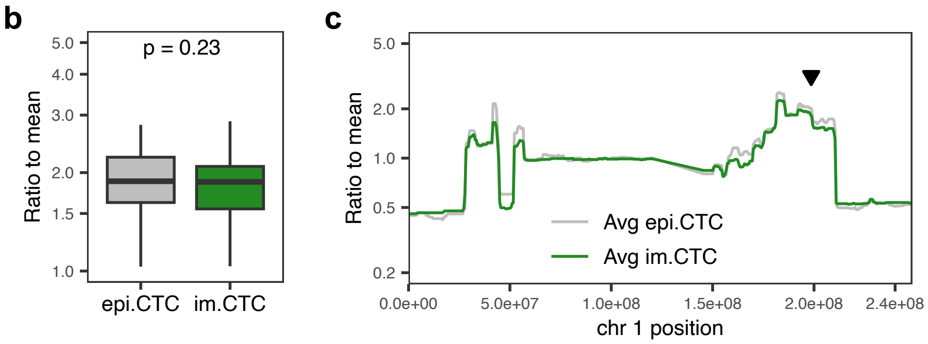

Supplementary Figure 3. CNAs on chromosome X and in the region of the *PTPRC* gene in im.CTCs versus epi.CTCs. (**a**) Distribution of epi.CTCs and im.CTCs with complete or partial loss of chromosome X. (**b**) Distribution of copy number ratios for the region on chromosome 1 containing the *PTPRC* gene for epi.CTCs and im.CTCs. Center line: median; box limits: 1st and 3rd quartiles; whiskers: 1.5x interquartile range. Two-sided Student's t-test p-value is also displayed. (**c**) Overlay of average epi.CTC and im.CTC copy number profiles for chromosome 1. Black arrow indicates the location of the *PTPRC* gene.

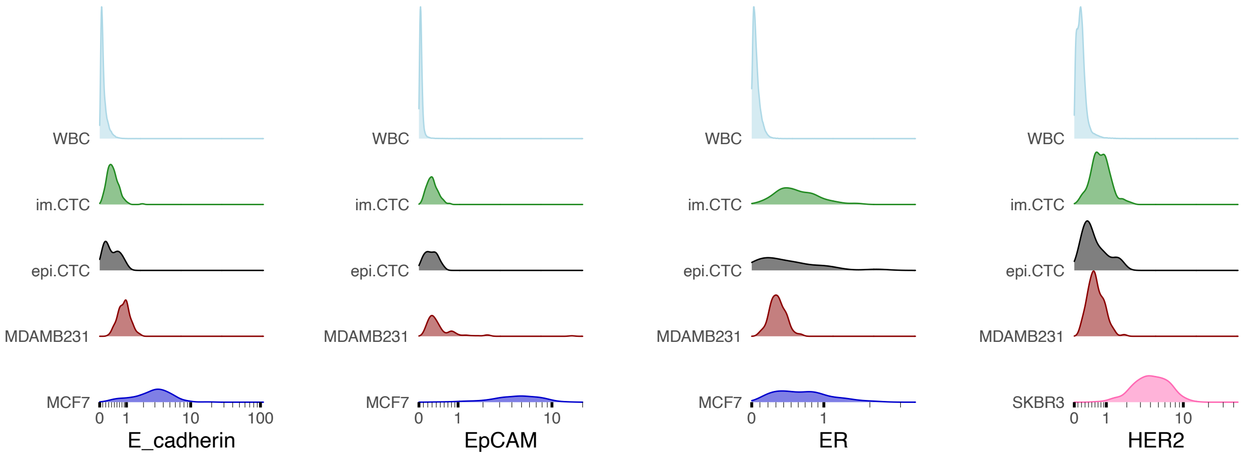

Supplementary Figure 4. Expression of breast cancer markers in index patient cells versus cell lines. Distribution of IMC ion counts for patient WBCs, im.CTCs, and epi.CTCs, and breast cancer cell lines with low and high expression of the indicated cancer markers. Ion counts are displayed with arcsinh-transformed x-axes.

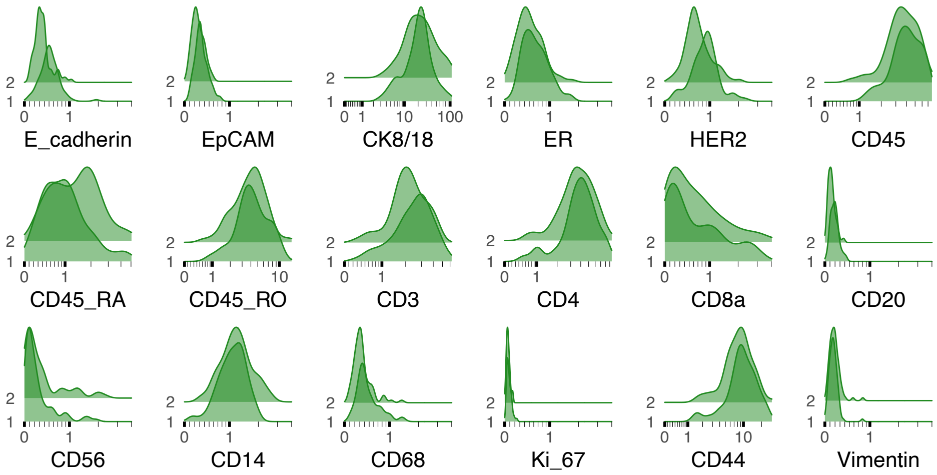

Supplementary Figure 5. Protein expression profiles in im.CTCs from pre- and post-treatment draws. Distributions of individual marker expression in im.CTCs from the first (n=94 cells) and second (n=78 cells) blood draws. Ion counts are displayed with arcsinh-transformed x-axes.

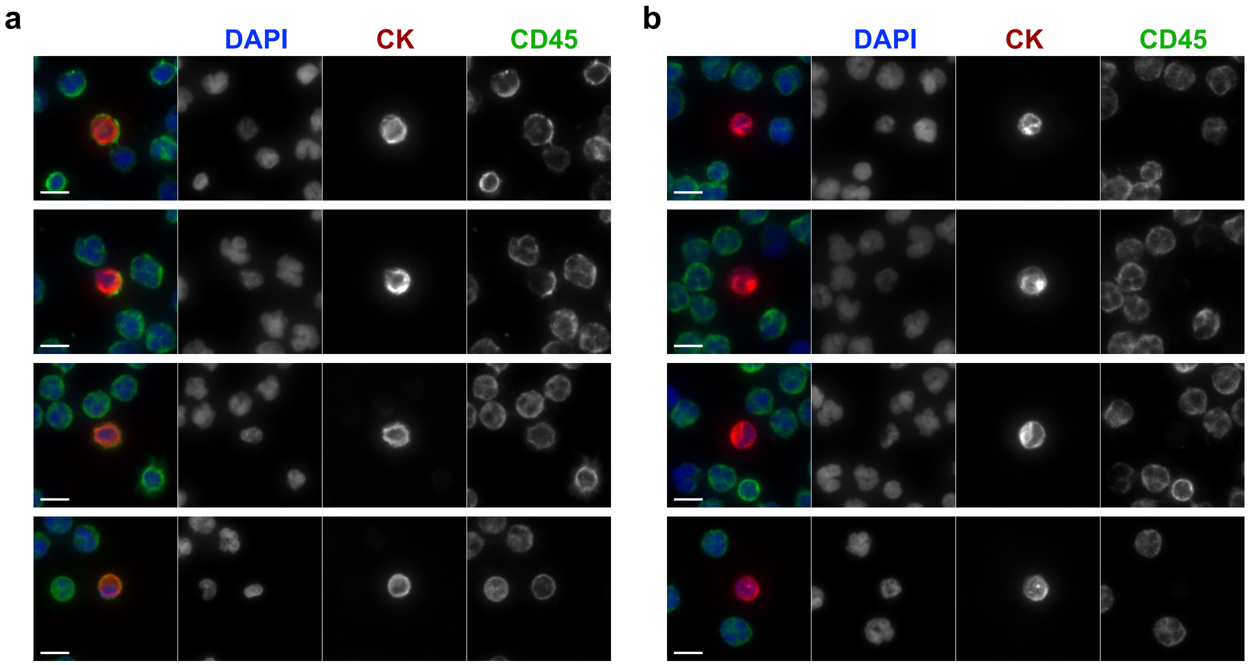

Supplementary Figure 6. CTC candidates from patient #2 and patient #3. Composite and individual channel images for (**a**) im.CTC and (**b**) epi.CTC candidates (scale bar=10µm).

Supplementary Table 1. Patient clinicopathological information and im.CTC status

	Receptor status	Histological subtype	im.CTCs detected
1	HR+/HER2-	Ductal	No
2	HR+/HER2-	Ductal	No
3	HR-/HER2-	Ductal	No
4	no data	Other	No
5	HR+/HER2-	Ductal	No
6	HR+/HER2-	Other	Yes (patient #2)
7	HR-/HER2-	Ductal	No
8	HR-/HER2-	Ductal	No
9	HR+/HER2-	Ductal	No
10	HR+/HER2-	Lobular	No
11	HR-/HER2-	Ductal	No
12	HR+/HER2-	Lobular	Yes (patient #1)
13	HR-/HER2-	Ductal	No
14	HR+/HER2-	Lobular	No
15	HR+/HER2-	Ductal	No
16	HR+/HER2-	Ductal	No
17	HR-/HER2-	Ductal	No
18	HR+/HER2-	Ductal	No
19	HR+/HER2-	Ductal	No
20	HR+/HER2-	Ductal	No
21	HR-/HER2+	Ductal	No
22	HR-/HER2-	Ductal	No
23	HR+/HER2-	Ductal	No
24	HR+/HER2-	Lobular	No
25	HR+/HER2-	Ductal	No
26	HR-/HER2-	Ductal	No
27	HR+/HER2+	Ductal	No
28	HR+/HER2-	Ductal	No
29	HR-/HER2-	Ductal	No
30	HR+/HER2-	Lobular	No
31	HR+/HER2+	Ductal	No
32	HR+/HER2+	Ductal	No
33	HR+/HER2-	Ductal	No
34	HR+/HER2-	Ductal	No
35	HR+/no HER2 data	Ductal	No
36	HR+/HER2-	Ductal	No
37	HR-/HER2-	Ductal	No

Supplementary Table 2. Panel of metal-tagged antibodies used for IMC experiments

Metal Tag	Target	Antibody Clone	Vendor	Cat#	Dilution (1:X)
169 Tm	CD66b	Rabbit_IgG_EPR20721	Abcam	ab229074	100
141 Pr	EpCAM	Mouse_IgG2b_9C4	Fluidigm	3141006B	200
161 Dy	Ki-67	Rabbit_IgG_EPR3610	Abcam	ab209897	200
166 Er	CD45-RA	Mouse_IgG2bk_HI100	Fluidigm	3166031D	200
209 Bi	CD16	Mouse_IgG1k_3G8	Fluidigm	3209002B	200
153 Eu	N-cadherin	Rabbit_IgG_SP90	Abcam	ab240403	200
158 Gd	E-cadherin	Rabbit_IgG_24E10	Fluidigm	3158029D	200
149 Sm	CD56	Mouse_IgG2b k_NCAM16.2	Fluidigm	3149021B	200
176 Yb	CD4	Rabbit_IgG_EPR6855	Abcam	ab181724	200
162 Dy	CD8a	Rabbit_IgG_D8A8Y	Fluidigm	3162035D	200
142 Nd	CD20	Rabbit_IgG_SP32	Abcam	ab236434	200
145 Nd	PR	Rabbit_IgG_YR85	Abcam	ab206926	200
174 Yb	HER2 (c-erbB2)	Mouse_IgG2b_42/c-erbB2	Fluidigm	3174021B	200
164 Dy	ER	Rabbit_IgG_D6R2W	Cell Signaling Technology	13258BF (custom order)	200
173 Yb	CD45-RO	Mouse_IgG2a_UCHL1	Fluidigm	3173016D	250
89 Y	CD45	Mouse_IgG1k_HI30	Fluidigm	3089003B	300
154 Sm	Vimentin	Rabbit IgG_EPR3776	Abcam	ab193555	300
170 Er	CD3	Rabbit_IgG_Polyclonal	Fluidigm	3170019D	400
175 Lu	CK8	Rabbit_IgG_EP1628Y	Abcam	ab217173	600
175 Lu	CK18	Rabbit_IgG_EPR1626	Abcam	ab240054	600
156 Gd	CD14	Rabbbit_IgG_EPR3653	Abcam	ab214438	600
159 Tb	CD68	Rabbit_IgG_EPR20545	Abcam	ab227458	600
171 Yb	CD44	Rat_IgG2b_IM7 (Helios)	Fluidigm	3171003B	1000
191 Ir	DNA1	Cell-ID^{TM} Intercalator	Fluidigm	NA	NA
193 Ir	DNA2	Cell-ID^{TM} Intercalator	Fluidigm	NA	NA
