# Supplementary material for: Simultaneous expression of epithelial and immune cell markers in circulating tumor cells identified in patients with stage 4 breast cancer
Source: Commun Med (Lond). 2025 Jul 24;5:309. doi: 10.1038/s43856-025-01024-0 (PMC12290075; doi:10.1038/s43856-025-01024-0)
Supplement: Supplementary file 2 — Reporting Summary [file 43856_2025_1024_MOESM2_ESM.pdf]

nature portfolio

Corresponding author(s): James Hicks, Peter Kuhn

Last updated by author(s): 2025 04 24

Reporting Summary

Nature Portfolio wishes to improve the reproducibility of the work that we publish. This form provides structure for consistency and transparency in reporting. For further information on Nature Portfolio policies, see our Editorial Policies and the Editorial Policy Checklist.

Statistics

For all statistical analyses, confirm that the following items are present in the figure legend, table legend, main text, or Methods section.

n/a	Confirmed	
☐	☒	The exact sample size (n) for each experimental group/condition, given as a discrete number and unit of measurement
☐	☒	A statement on whether measurements were taken from distinct samples or whether the same sample was measured repeatedly
☐	☒	The statistical test(s) used AND whether they are one- or two-sided<br>*Only common tests should be described solely by name; describe more complex techniques in the Methods section.*
☒	☐	A description of all covariates tested
☒	☐	A description of any assumptions or corrections, such as tests of normality and adjustment for multiple comparisons
☒	☐	A full description of the statistical parameters including central tendency (e.g. means) or other basic estimates (e.g. regression coefficient) AND variation (e.g. standard deviation) or associated estimates of uncertainty (e.g. confidence intervals)
☒	☐	For null hypothesis testing, the test statistic (e.g. F, t, r) with confidence intervals, effect sizes, degrees of freedom and P value noted<br>*Give P values as exact values whenever suitable.*
☒	☐	For Bayesian analysis, information on the choice of priors and Markov chain Monte Carlo settings
☒	☐	For hierarchical and complex designs, identification of the appropriate level for tests and full reporting of outcomes
☒	☐	Estimates of effect sizes (e.g. Cohen's d, Pearson's r), indicating how they were calculated

Our web collection on statistics for biologists contains articles on many of the points above.

Software and code

Policy information about availability of computer code

Data collection	The code used in this study utilizes standard third-party open-source libraries packaged in R and Python. The image analysis code used to identify rare cell candidates in the HDSCA workflow uses custom code and is proprietary and licensed to Epic Sciences for commercial use.
Data analysis	Data visualization and statistical testing was performed in R v4.1.2. Two-sided Student's t-test was used to compare PTPRC copy number ratios between im.CTCs and epi.CTCs. Plots were created with the ggplot2 v3.3.6, ComplexHeatmap v2.10.0, and umap v0.2.10.0 packages.

For manuscripts utilizing custom algorithms or software that are central to the research but not yet described in published literature, software must be made available to editors and reviewers. We strongly encourage code deposition in a community repository (e.g. GitHub). See the Nature Portfolio guidelines for submitting code & software for further information.

Data

Policy information about availability of data

All manuscripts must include a data availability statement. This statement should provide the following information, where applicable:

- Accession codes, unique identifiers, or web links for publicly available datasets
- A description of any restrictions on data availability
- For clinical datasets or third party data, please ensure that the statement adheres to our policy

The data presented in this study are available through the BloodPAC Commons https://data.bloodpac.org/discovery/BPDC000144.

Research involving human participants, their data, or biological material

Policy information about studies with human participants or human data. See also policy information about sex, gender (identity/presentation), and sexual orientation and race, ethnicity and racism.

Reporting on sex and gender	The sex of the case study patient was reported but no sex or gender analyses were performed due to the small sample size.
Reporting on race, ethnicity, or other socially relevant groupings	Race data was not collected from the case study patient and was not reported.
Population characteristics	The case study patient was a female patient diagnosed with estrogen receptor (ER) positive, HER2 negative breast cancer that had metastasized to the skin and bone. Patients included in the cohort analysis were enrolled from the same BloodPAC-007 study as the case study patient. This study enrolled breast cancer patients aged 18 or older with metastatic disease who were starting a new line of therapy at the Walter Reed National Military Medical Center Murtha Cancer Center (WRNMMC MCC) or Anne Arundel Medical Center (AAMC).
Recruitment	The case study patient was enrolled as part of the BloodPAC-007 study. This study enrolled breast cancer patients aged 18 or older with metastatic disease who were starting a new line of therapy at the Walter Reed National Military Medical Center Murtha Cancer Center (WRNMMC MCC) or Anne Arundel Medical Center (AAMC).
Ethics oversight	This study was approved by the Institutional Review Board (or Ethics Committee) of the WRNMMC (WRNMMC-2018-0130) AAMC (AAMC-1109045), and University of Southern California (UP-17-00882), and adhered to the principles in the Declaration of Helsinki. Written informed consent was obtained from the patient in this study.

Note that full information on the approval of the study protocol must also be provided in the manuscript.

Field-specific reporting

Please select the one below that is the best fit for your research. If you are not sure, read the appropriate sections before making your selection.

☒ Life sciences ☐ Behavioural & social sciences ☐ Ecological, evolutionary & environmental sciences

For a reference copy of the document with all sections, see nature.com/documents/nr-reporting-summary-flat.pdf

Life sciences study design

All studies must disclose on these points even when the disclosure is negative.

Sample size	For the index case, two peripheral blood draws from one patient with metastatic breast cancer were analyzed. For the cohort analysis, 36 additional peripheral blood samples from 36 additional metastatic breast cancer patients were analyzed.
Data exclusions	No data was excluded.
Replication	Replicates were not able to be performed due to the nature of the patient-derived blood samples.
Randomization	This is not relevant to our study. This is not relevant to our study. This was an observational study on the characteristics of CD45+ circulating tumor cells.
Blinding	This is not relevant to our study. This was an observational study on the characteristics of CD45+ circulating tumor cells.

Reporting for specific materials, systems and methods

We require information from authors about some types of materials, experimental systems and methods used in many studies. Here, indicate whether each material, system or method listed is relevant to your study. If you are not sure if a list item applies to your research, read the appropriate section before selecting a response.

Materials & experimental systems

n/a	Involved in the study
☐	☒ Antibodies
☒	☐ Eukaryotic cell lines
☒	☐ Palaeontology and archaeology
☒	☐ Animals and other organisms
☐	☒ Clinical data
☒	☐ Dual use research of concern
☒	☐ Plants

Methods

n/a	Involved in the study
☒	☐ ChIP-seq
☒	☐ Flow cytometry
☒	☐ MRI-based neuroimaging

Antibodies

Antibodies used

Immunofluoresence staining: anti-human CK 1,4,5,6,8,10,13,18,19 mouse IgG1/IgG2a monoclonal antibody cocktail (Sigma; Cat# C2562; Clones: C-11, PCK-26, CY-90, KS-1A3, M20, A53-B/A2), anti-human CK 19 mouse IgG1 monoclonal antibody (Dako; Cat# GA61561–2; Clone: RCK108), anti-human CD45:Alexa Fluor 647 mouse IgG2a monoclonal antibody (AbD Serotec; Cat# MCA87A647; Clone: F10–89–4), anti-human CD41 rabbit IgG polyclonal antibody (Invitrogen; Cat# PA522307), anti-mouse IgG1:Alexa Fluor 555 goat IgG polyclonal antibody (Invitrogen; Cat# A21127), anti-rabbit IgG:Alexa Fluor 488 goat IgG polyclonal antibody (Abcam; Cat# ab150077). Imaging mass cytometry: CD66b (Abcam; Cat# ab229074; Clone: Rabbit_IgG_EPR20721), EpCAM (Fluidigm; Cat# 3141006B; Clone: Mouse_IgG2b_9C4), Ki-67 (Abcam; Cat# ab209897; Clone: Rabbit_IgG_EPR3610), CD45-RA (Fluidigm; Cat# 3166031D; Clone: Mouse_IgG2bk_HI100), CD16 (Fluidigm; Cat# 3209002B; Clone: Mouse_IgG1k_3G8), N-cadherin (Abcam; Cat# ab240403; Clone: Rabbit_IgG_SP90), E-cadherin (Fluidigm; Cat# 3158029D; Clone: Rabbit_IgG_24E10), CD56 (Fluidigm; Cat# 3149021B; Clone: Mouse_IgG2b k_NCAM16.2), CD4 (Abcam; Cat# ab181724; Clone: Rabbit_IgG_EPR6855), CD8a (Fluidigm; Cat# 3162035D; Clone: Rabbit_IgG_D8A8Y), CD20 (Abcam; Cat# ab236434; Clone: Rabbit_IgG_SP32), PR (Abcam; Cat# ab206926; Clone: Rabbit_IgG_YR85), HER2 (c-erbB2) (Fluidigm; Cat# 3174021B; Clone: Mouse_IgG2b_42/c-erbB2), ER (Cell Signaling Technology; Cat# 13258BF (custom order); Clone: Rabbit_IgG_D6R2W), CD45-RO (Fluidigm; Cat# 3173016D; Clone: Mouse_IgG2a_UCHL1), CD45 (Fluidigm; Cat# 3089003B; Clone: Mouse_IgG1k_HI30), Vimentin (Abcam; Cat# ab193555; Clone: Rabbit IgG_EPR3776), CD3 (Fluidigm; Cat# 3170019D; Clone: Rabbit_IgG_Polyclonal), CK8 (Abcam; Cat# ab217173; Clone: Rabbit_IgG_EP1628Y), CK18 (Abcam; Cat# ab240054; Clone: Rabbit_IgG_EPR1626), CD14 (Abcam; Cat# ab214438; Clone: Rabbbit_IgG_EPR3653), CD68 (Abcam; Cat# ab227458; Clone: Rabbit_IgG_EPR20545), CD44 (Fluidigm; Cat# 3171003B; Clone: Rat_IgG2b_IM7 (Helios))

Validation

Overall performance, including sensitivity and specificity, in both healthy donors and cancer patients was conducted as described previously with references provided in the text.

Clinical data

Policy information about clinical studies

All manuscripts should comply with the ICMJE guidelines for publication of clinical research and a completed CONSORT checklist must be included with all submissions.

Clinical trial registration

Samples collected from patients at Anne Arundel Medical Center (AAMC-1109045) and Walter Reed National Military Medical Center (WRNMMC-2018-0130).

Study protocol

Full study protocol is available upon request.

Data collection

Data was collected during the recruitment and treatment period and provided as de-identified information in a secure database.

Outcomes

This was an observational study without pre-defined outcome measures.

Plants

Seed stocks

Report on the source of all seed stocks or other plant material used. If applicable, state the seed stock centre and catalogue number. If plant specimens were collected from the field, describe the collection location, date and sampling procedures.

Novel plant genotypes

Describe the methods by which all novel plant genotypes were produced. This includes those generated by transgenic approaches, gene editing, chemical/radiation-based mutagenesis and hybridization. For transgenic lines, describe the transformation method, the number of independent lines analyzed and the generation upon which experiments were performed. For gene-edited lines, describe the editor used, the endogenous sequence targeted for editing, the targeting guide RNA sequence (if applicable) and how the editor was applied.

Authentication

Describe any authentication procedures for each seed stock used or novel genotype generated. Describe any experiments used to assess the effect of a mutation and, where applicable, how potential secondary effects (e.g. second site T-DNA insertions, mosiacism, off-target gene editing) were examined.
